# A Residual-Based Mathematical Approach to Evaluate Production-Adjusted Nitrogen Use Efficiency and Metabolic Responses in Dairy Cows

**DOI:** 10.3390/vetsci13070637

**Published:** 2026-06-30

**Authors:** Yunfei Zhai, Jiaxuan Song, Hantong Weng, Haihui Wang, Tianqin Hu, Zhaoyu Han

**Affiliations:** 1 Institute of Dairy Science, College of Animal Science and Technology, Nanjing Agricultural University, Nanjing 210095, China; 2022205004@stu.njau.edu.cn (Y.Z.); 2024805106@stu.njau.edu.cn (J.S.); 2022105011@stu.njau.edu.cn (H.W.); 2022105001@stu.njau.edu.cn (H.W.); 2 Technical Promotion Department, Qinghai Provincial Animal Genetic Resources Protection and Utilization Center, Xining 810000, China; qhxbhtq@163.com

**Keywords:** nitrogen use efficiency, residual NUE, mathematical modeling, dairy cows, rumen fermentation, amino acid metabolism, metabolic efficiency

## Abstract

Nitrogen use efficiency (NUE) is commonly calculated as the ratio of milk nitrogen output to nitrogen intake in dairy cows. However, because milk nitrogen output is intrinsically determined by milk production and nitrogen intake is largely driven by dry matter intake, conventional NUE is mathematically dependent on production level and feed intake. This dependency makes it difficult to distinguish apparent efficiency caused by higher milk yield from intrinsic biological efficiency in nitrogen utilization. Therefore, this study aimed to evaluate NUE in dairy cows using a combined mathematical and metabolic framework based on residual NUE (rNUE) analysis. A total of 126 early-lactation Chinese Holstein cows were screened, and 16 high-NUE and 16 low-NUE cows were selected after matching for parity, days in milk, and dry matter intake. High-NUE cows had greater milk yield, milk nitrogen output, and NUE than low-NUE cows, despite similar nitrogen intake. They also exhibited higher ruminal microbial crude protein and ammonia nitrogen concentrations, a lower acetate-to-propionate ratio, and reduced circulating essential and total amino acid concentrations. Multiple regression analysis showed that energy-corrected milk and dry matter intake explained 71.4% of the variation in NUE. The residuals from this model were defined as rNUE, which was independent of milk production. After removing production-related effects, rNUE remained positively associated with ruminal microbial crude protein and ammonia nitrogen concentrations, and negatively associated with the acetate-to-propionate ratio and circulating amino acid pools. These findings indicate that conventional NUE in dairy cows is largely driven by production level, whereas residual-based modeling can identify a production-independent component associated with rumen nitrogen metabolism and amino acid utilization. The residual NUE approach provides a useful mathematical and metabolic framework for evaluating intrinsic NUE in dairy cows beyond milk production level.

## Simple Summary

Feeding dairy cows efficiently is important both for farm profitability and for reducing environmental pollution. Cows need dietary nitrogen (a nutrient found in protein) to produce milk, but much of this nitrogen is wasted through manure. A common way to measure nitrogen efficiency is to compare the amount of nitrogen in milk to the amount eaten. However, this measurement is heavily influenced by how much milk a cow produces, making it difficult to tell whether a cow is truly efficient at using nitrogen or simply producing a lot of milk. In this study, we used a mathematical method to separate the effect of milk production from the cow’s own ability to use nitrogen. We found that most of the variation in conventional nitrogen efficiency was explained by milk yield and feed intake. After removing these production effects, the remaining efficiency was linked to better rumen function and more effective use of amino acids in the body. This new way of measuring efficiency could help farmers and breeders identify cows that are naturally better at using dietary nitrogen, which may reduce feed costs and nitrogen pollution from dairy farming.

## 1. Introduction

In recent decades, advances in genetic selection and feeding management have markedly increased milk yield in dairy cows; however, nitrogen use efficiency (NUE) remains relatively low [1]. In the present study, NUE refers specifically to milk nitrogen efficiency, calculated as milk nitrogen output divided by nitrogen intake, rather than total nitrogen conversion or actual whole-body nitrogen retention. Across lactating dairy cows, approximately 20–35% of dietary nitrogen is typically recovered as milk nitrogen, whereas most dietary nitrogen is excreted via feces and urine [2,3,4]. However, this proportion varies with lactation stage, breed, dietary crude protein concentration, protein degradability, and feeding management [5,6]. Studies in early-lactation Holstein cows and Holstein-Friesian cows at different lactation stages have further shown that dietary crude protein concentration can affect milk nitrogen efficiency, urinary nitrogen excretion, and overall nitrogen partitioning [5,7,8]. Such inefficient nitrogen utilization not only increases feeding costs, especially those associated with protein supplements, but also aggravates environmental risks, including ammonia volatilization and nitrate leaching [9,10]. Therefore, improving NUE has become a key goal for enhancing the sustainability of modern dairy production systems [11].

Most strategies to improve NUE have focused on nutritional manipulation, such as optimizing dietary crude protein levels [12,13,14], balancing rumen-degradable and rumen-undegradable protein [15,16,17], improving energy–nitrogen synchrony [18,19,20], and applying feed additives [21,22,23]. Although these approaches can partially enhance nitrogen utilization [13,15,16], substantial inter-individual variation in NUE persists among cows managed under similar feeding and husbandry conditions [24,25,26]. This persistent variability suggests that, beyond dietary factors, inherent physiological and metabolic characteristics of the cow may play an important role in determining NUE [27,28].

However, a critical limitation in current NUE research is that conventional NUE is mathematically dependent on milk production and feed intake [3,29]. Because NUE is calculated as the ratio of milk nitrogen output to nitrogen intake, milk nitrogen output is largely determined by milk yield and milk protein secretion, whereas nitrogen intake is mainly driven by dry matter intake and dietary nitrogen concentration. As a result, cows with higher milk production may exhibit higher apparent NUE even when their intrinsic metabolic efficiency is not necessarily improved. This mathematical dependency makes it difficult to distinguish production-driven differences from true biological variation in nitrogen utilization. Therefore, improving the evaluation of nutritional efficiency requires not only dietary interventions but also appropriate analytical strategies that can separate production-related and production-independent components of NUE.

Residual-based analytical strategies may provide a useful framework to address this limitation. This approach is conceptually analogous to residual feed intake (RFI), a widely used phenotype in ruminant nutrition and breeding research, which is commonly defined as the difference between observed feed intake and feed intake predicted from maintenance and production requirements [30,31,32]. In statistical terms, a residual represents the deviation between an observed value and the value predicted by a regression model. Applying this principle to nitrogen utilization, residual NUE (rNUE) can be calculated as the difference between observed NUE and NUE predicted from major production-related factors, such as ECM and dry matter intake (DMI). Compared with conventional NUE, which is inherently influenced by milk nitrogen output and nitrogen intake, rNUE statistically strips out the variation in NUE explained by ECM and DMI. Therefore, rNUE may provide a complementary phenotype for evaluating NUE that is less confounded by production level and feed intake. Such an approach could provide a more objective basis for evaluating intrinsic metabolic efficiency in dairy cows and for identifying biological mechanisms underlying individual differences in nitrogen utilization.

In recent years, increasing attention has been directed toward the biological basis of individual differences in NUE [33,34]. Previous studies have reported that cows with higher NUE often exhibit greater milk protein output and lower urea-related indicators, such as milk urea nitrogen and serum urea nitrogen [16,25,35]. However, several important gaps remain. First, because conventional NUE is calculated from milk nitrogen output and nitrogen intake, many comparisons based on conventional NUE may still be confounded by milk production level and feed intake, making it difficult to distinguish production-driven apparent efficiency from production-adjusted biological variation in nitrogen utilization. Second, although nitrogen excretion has been widely evaluated, fewer studies have simultaneously characterized nitrogen partitioning among milk nitrogen output, fecal nitrogen, urinary nitrogen, and apparent nitrogen balance when intake and lactation stage are statistically accounted for. Third, rumen fermentation, circulating amino acid availability, and milk amino acid composition are often examined separately, and an integrated assessment of the rumen–blood amino acid–mammary gland axis in relation to divergent NUE remains limited. Therefore, a framework combining residual-based adjustment of NUE with nitrogen partitioning and metabolic profiling may help clarify production-adjusted variation in nitrogen utilization among dairy cows.

Therefore, this study aimed to evaluate nitrogen use efficiency in early-lactation dairy cows using a residual-based mathematical approach combined with metabolic profiling. Specifically, the first objective was to quantify the extent to which conventional NUE was explained by major production-related factors, including ECM and DMI. The second objective was to derive rNUE, defined as the deviation of observed NUE from NUE predicted by ECM and DMI, as a production-adjusted phenotype for evaluating individual differences in nitrogen utilization. The third objective was to relate rNUE to nitrogen partitioning, rumen fermentation characteristics, serum amino acid profiles, and milk amino acid composition, in order to identify metabolic features associated with production-adjusted NUE. We hypothesized that conventional NUE would be strongly associated with milk production and feed intake, whereas rNUE would capture biologically meaningful variation less confounded by production level and would be associated with ruminal nitrogen metabolism and amino acid utilization.

## 2. Materials and Methods

### 2.1. Ethical Statement

All procedures used in the present study complied with the laws and regulations of China and the internationally accepted principles and guidelines for the care and use of agricultural, laboratory, or experimental animals. The animal study protocol was approved by the Experimental Animal Welfare and Ethics Committee of Nanjing Agricultural University, China (approval code: SYXK (Su) 2017–0027; approval date: 7 December 2017).

### 2.2. Animals, Experimental Design, and Feeding Management

The experiment was conducted in April 2023 at a commercial dairy farm located in Jiangsu Province, China. The farm maintained a total of 559 lactating cows. Among them, 126 early-lactation Holstein cows (≤100 days in milk) were initially enrolled as candidate animals for NUE evaluation. Holstein cows were selected because they are the predominant high-yielding dairy breed used in commercial milk production systems in China and therefore provide a relevant population for evaluating individual variation in NUE under practical Chinese dairy production conditions.

All cows were housed in a tie-stall barn and fed a total mixed ration (TMR) formulated according to the Chinese Feeding Standards for Dairy Cattle. Feed was offered three times daily, and cows had ad libitum access to feed and water throughout the experimental period. Cows were milked three times daily. The ingredient composition and nutrient levels of the experimental diet are presented in Table 1.

The experimental period consisted of an initial 37 d screening phase followed by a 7 d intensive sampling phase. No additional adaptation period was imposed before the screening phase because this was an observational extreme-phenotype study rather than a dietary or housing intervention trial. All candidate cows were selected from the resident herd of the same commercial farm and had already been maintained under the same housing conditions and fed the same TMR before enrollment. Therefore, the screening phase was designed to characterize baseline individual variation in NUE under stable management conditions rather than to serve as an adaptation period to a new treatment. During the 37 d screening phase, individual DMI and milk yield were recorded repeatedly, and milk composition data were used to calculate milk nitrogen output and conventional NUE. This phase had two main purposes: first, to obtain repeated measurements for reducing short-term fluctuations in DMI, milk production, and NUE; and second, to identify cows with divergent NUE phenotypes under comparable feeding and management conditions.

To reduce potential confounding effects associated with early lactation and health status, parity, days in milk, DMI, and available health records were considered during cow selection. Cows with clinical mastitis or other recorded diseases during the screening or sampling period were excluded from the candidate population. Somatic cell count was recorded as an udder-health-related indicator and compared between groups, but it was not used as a primary criterion for defining high- or low-NUE phenotypes. During the 37 d screening phase, individual NUE was calculated from repeated measurements and cows were ranked according to their average NUE. Cows in approximately the upper 20% and lower 20% of the NUE distribution were considered high- and low-NUE candidates, respectively. From these candidates, cows were further selected to maintain comparable parity, days in milk, and DMI between groups. Based on this intake- and lactation-stage-matched extreme-phenotype selection approach, 32 cows were classified into the high-NUE and low-NUE groups (*n* = 16 per group). Because this was an observational extreme-phenotype comparison rather than a randomized dietary or management intervention trial, cows were classified according to predefined NUE ranking and matching criteria rather than randomly assigned to treatments.

### 2.3. Measurements, Sample Collection, and Laboratory Analyses

During the 7 d consecutive sampling period, milk yield was recorded daily for each cow, and milk samples were collected at each milking. Milk samples from the morning, afternoon, and evening milkings were pooled proportionally according to the corresponding milk yield to obtain a representative daily composite sample. One aliquot of the composite milk sample was used for milk composition analysis, and another aliquot was immediately frozen in liquid nitrogen and stored at −80 °C for subsequent determination of hydrolyzed AA.

DMI was measured daily for each cow using individual feeding boxes. The TMR offered to each cow was weighed at each feeding, and feed refusals were collected and weighed before the subsequent feeding. Daily DMI was calculated as the difference between feed offered and feed refused on a dry matter basis. Samples of the TMR were collected daily during the sampling period and stored at −20 °C for subsequent analysis of diet composition and nutrient contents.

Fecal samples were collected using the total fecal collection method throughout the sampling period. Urine samples were collected according to the method described by H. Boudra et al., using spot urine sampling [37]. Body weight was estimated based on body measurements, including heart girth and body length, using a predictive equation in which body weight was calculated from the square of heart girth multiplied by body length and divided by a constant value of 10,840.

On the final day of the sampling period, before the morning feeding, blood samples were collected from the coccygeal vein into vacuum tubes. Samples were centrifuged at 3000× *g* for 15 min to obtain serum, which was then stored at −20 °C until analysis. Serum biochemical parameters were analyzed using an automated biochemical analyzer (AV400, Olympus Diagnostics, Tokyo, Japan) based on enzymatic colorimetric methods. Commercial assay kits were purchased from Nanjing Jiancheng Bioengineering Institute (Nanjing, China; catalog numbers: A045-2-2, A028-2-1, C013-2-1, A154-1-1, A110-1-1, A111-1-1, A020-2-2, and A015-2-1). All assays were conducted according to the manufacturers’ instructions, and serum samples were analyzed in triplicate.

Amino acid concentrations were determined using an amino acid analyzer (LA8080, Hitachi, Tokyo, Japan). Hydrolyzed AAs in milk samples were analyzed using an acid hydrolysis method, whereas free AAs in serum were determined after deproteinization of serum with sulfosalicylic acid at a volume ratio of 1:3 (serum: sulfosalicylic acid).

Rumen fluid samples were collected from a subset of cows (*n* = 8 per group) using an oral stomach tube. The subset cows were selected to represent the average characteristics of the high- and low-NUE groups in terms of DMI, days in milk, and lactation performance. Rumen fluid was collected once from each cow on the same day at approximately 3 h after the morning feeding (between 10:00 and 12:00), because this time point corresponds to the postprandial peak fermentation period and is commonly used for evaluating rumen fermentation status, and cows from the high- and low-NUE groups were sampled alternately to minimize systematic time-related bias between groups. For each cow, the oral stomach tube was inserted to an approximate depth of 180–200 cm to reach the rumen. The first 100 mL of rumen fluid was discarded to minimize potential saliva contamination. The collected rumen fluid was filtered through four layers of cheesecloth, and pH was measured immediately on site using a portable pH meter (Orion Star A121, Thermo Scientific, Waltham, MA, USA). Filtered rumen fluid samples were then frozen in liquid nitrogen and stored for subsequent analysis of rumen fermentation parameters.

Volatile fatty acid (VFA) concentrations were determined using a gas chromatograph (GC-2014, Shimadzu, Kyoto, Japan) equipped with a capillary column, following the method described by Hu et al. [38]. Microbial crude protein (MCP) concentration was quantified using the Coomassie Brilliant Blue G-250 staining method. NH_3_-N concentration was determined according to the method described by Thiex et al. [39].

### 2.4. Nitrogen Partitioning and Calculations

Nitrogen intake (N intake) was calculated based on DMI and the nitrogen concentration of the diet. Dietary nitrogen concentration was obtained by dividing dietary crude protein content by 6.25. Milk nitrogen (milk N) output was calculated using daily milk yield and milk protein concentration, with milk protein converted to nitrogen using a factor of 6.38. Fecal nitrogen (fecal N) was determined based on fecal dry matter output and fecal nitrogen concentration. Urinary nitrogen (urinary N) excretion was determined according to the method described by Doran et al. [40]. Apparent nitrogen balance was calculated by difference using the following equation:

$$Apparent\;N\;balance=N_{intake}-N_{milk}-N_{feces}-N_{urine}$$

Because gaseous nitrogen losses, such as ammonia volatilization, and other unmeasured nitrogen loss pathways were not quantified in the present study, apparent N balance was interpreted as the fraction of nitrogen not accounted for by the measured output pathways rather than as direct evidence of actual tissue nitrogen deposition or whole-body nitrogen retention. NUE was calculated as the proportion of nitrogen intake secreted in milk. All nitrogen variables were expressed on a daily basis.

### 2.5. Statistical Analysis

All data were first organized using Microsoft Excel (Microsoft Excel 2019, Microsoft Corporation, Redmond, WA, USA). Statistical analyses were performed using SPSS software (version 26.0; IBM Corp., Armonk, NY, USA). Data are presented as means ± SEM. Prior to analysis, data normality was assessed using the Shapiro–Wilk test, and homogeneity of variance was evaluated using Levene’s test. For variables that were normally distributed and showed homogeneous variances, differences between the high- and low-NUE groups were analyzed using an independent-samples *t*-test. For normally distributed variables with unequal variances, Welch’s *t*-test was applied. Variables that did not meet the assumption of normality were analyzed using the Mann–Whitney U test. All tests were two-tailed. Differences were considered statistically significant at *p* < 0.05, whereas 0.05 ≤ *p* < 0.10 was considered indicative of a tendency toward statistical significance.

To account for the influence of production level and feed intake on conventional NUE, residual analysis was performed using the full screened population of cows. Conventional NUE was used as the dependent variable, and ECM and DMI were included as independent variables in a multiple linear regression model, as follows:

$$NUE_{i}=\beta_{0}+\beta_{1}\times ECM_{i}+\beta_{2}\times DMI_{i}+{\;\varepsilon }_{i}$$

where ε_i_ represents the residual term for the ith cow. The unstandardized residuals from this model were extracted and defined as residual NUE (rNUE). Positive rNUE values indicate that a cow had a higher NUE than expected based on its ECM and DMI, whereas negative values indicate a lower NUE than expected. Unstandardized residuals were used because they retain the original scale of NUE and therefore allow direct interpretation of the magnitude and direction of deviation from predicted NUE. Before regression analysis, the dataset was checked for completeness and potential data entry errors. Variables were retained on their original scale to preserve biological interpretability. Model goodness of fit was evaluated using the coefficient of determination (R^2^), adjusted R^2^, and the overall F-test. Residual diagnostics were performed by assessing the distribution of unstandardized residuals using the Kolmogorov–Smirnov test with Lilliefors correction and visual inspection of normal Q-Q plots. Standardized residuals were also examined to identify potential outlying observations. A sensitivity analysis was conducted by excluding the observation with the most extreme standardized residual to evaluate the robustness of the regression model. Multicollinearity among independent variables was evaluated using tolerance and variance inflation factor (VIF). In subsequent linear regression analyses examining associations between rNUE and rumen fermentation parameters, serum amino acid concentrations, and biochemical indicators, standardized regression coefficients (β), R^2^, and *p*-values were reported. R^2^ was used to indicate the proportion of variance explained by each model, and *p*-values were used to determine statistical significance.

## 3. Results

### 3.1. Body Measurement Traits

The comparison of body measurement traits between cows with high and low NUE is presented in Table 2. No significant differences were observed between the two groups in body height, chest circumference, or body weight (*p* > 0.05). Cows in the low-NUE group tended to have a greater body length compared with those in the high-NUE group; however, this difference did not reach statistical significance (*p* = 0.088).

### 3.2. Lactation Performance and Its Association with Nitrogen Use Efficiency

**Figure 1:**
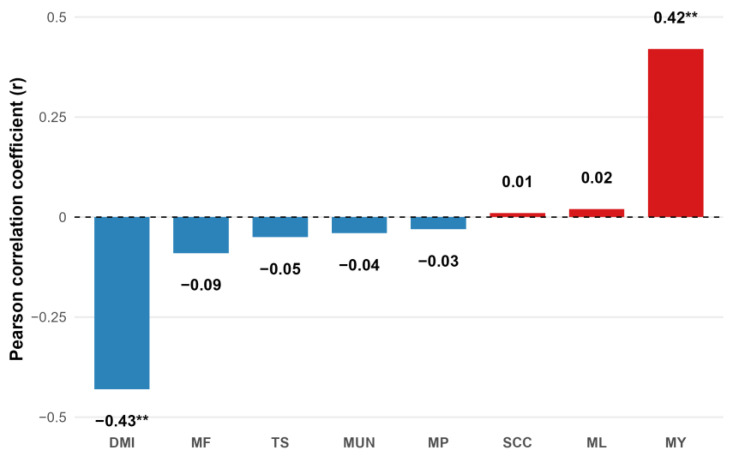
Pearson correlations between nitrogen use efficiency and production traits based on individual cow data (*n* = 126). Values are Pearson correlation coefficients (r). DMI—dry matter intake; MY—milk yield; MF—milk fat concentration; MP—milk protein concentration; ML—milk lactose concentration; TS—total solids; MUN—milk urea nitrogen; SCC—somatic cell count. ** *p* < 0.01.

Pearson correlation analysis based on data from all 126 cows showed that NUE was negatively correlated with DMI (r = −0.430, *p* < 0.01) and positively correlated with milk yield (r = 0.420, *p* < 0.01). No significant correlations were observed between NUE and milk composition traits, milk urea nitrogen, or somatic cell count (Figure 1).

The lactation performance of cows with high and low NUE is summarized in Table 3. Parity, days in milk, and DMI did not differ significantly between the two groups (*p* > 0.05). Cows in the high-NUE group exhibited a significantly greater milk yield compared with those in the low-NUE group (*p* < 0.001). Milk protein concentration was significantly lower in the high-NUE group than in the low-NUE group (*p* < 0.001), whereas milk protein yield was significantly greater in cows with high NUE (*p* < 0.001). Milk fat concentration was also significantly lower in the high-NUE group (*p* < 0.001), and milk fat yield showed a tendency to be higher in the high-NUE group compared with the low-NUE group (*p* = 0.081). Lactose concentration was slightly but significantly lower in the high-NUE group compared with the low-NUE group (*p* = 0.014). Total solids concentration, milk urea nitrogen, and the fat-to-protein ratio were all significantly lower in cows with high NUE than in those with low NUE (*p* < 0.01). No significant difference was observed in somatic cell count between the two groups (*p* > 0.05).

### 3.3. Rumen Fermentation Parameters

The rumen fermentation parameters of cows with high and low NUE are presented in Table 4. The concentration of MCP was significantly greater in the high-NUE group than in the low-NUE group (*p* < 0.001). Rumen pH did not differ significantly between the two groups (*p* > 0.05). The concentration of ruminal NH_3_-N was significantly higher in cows with high NUE compared with those with low NUE (*p* = 0.009). No significant differences were observed between the two groups in acetate concentration, isobutyric acid, isovaleric acid, butyrate, valeric acid, or total volatile fatty acid concentration (*p* > 0.05). Propionate concentration tended to be higher in the H group compared with the L group (*p* = 0.062). The acetate-to-propionate ratio was significantly lower in the high-NUE group than in the low-NUE group (*p* = 0.042).

### 3.4. Biochemical Parameters

**Figure 2:**
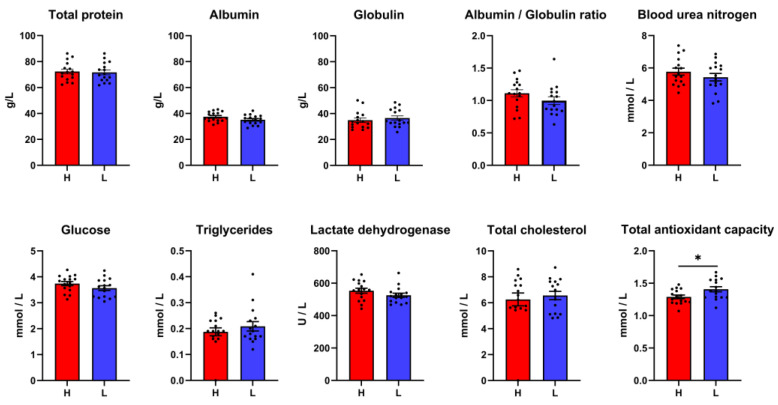
Comparison of biochemical parameters between cows with high and low nitrogen use efficiency. * *p* < 0.05.

The biochemical parameters of cows with high and low NUE are presented in Figure 2. No significant differences were observed between the two groups in total protein, globulin concentration, albumin-to-globulin ratio, serum urea nitrogen, glucose, total cholesterol, triglycerides, or lactate dehydrogenase activity (*p* > 0.05). Albumin concentration tended to be higher in the high-NUE group compared with the low-NUE group, although this difference did not reach statistical significance (*p* = 0.090). Total antioxidant capacity was significantly lower in cows with high NUE than in those with low NUE (*p* = 0.020).

### 3.5. Nitrogen Partitioning

Comparisons of nitrogen partitioning between cows with high and low NUE are shown in Table 5. Nitrogen intake did not differ significantly between the two groups (*p* > 0.05). Milk nitrogen output was significantly greater in the high-NUE group than in the low-NUE group (*p* < 0.001). Accordingly, NUE was significantly higher in cows with high NUE compared with those with low NUE (*p* < 0.001). Urinary nitrogen excretion, expressed both as absolute amount and as a proportion of nitrogen intake, was significantly higher in the high-NUE group than in the low-NUE group (*p* < 0.05). In contrast, fecal nitrogen excretion, either in absolute amount or as a percentage of nitrogen intake, did not differ significantly between the two groups (*p* > 0.05). Apparent nitrogen balance was significantly lower in cows with high NUE compared with those with low NUE (*p* < 0.001).

### 3.6. Serum-Free Amino Acid Concentrations

Comparisons of serum-free amino acid concentrations between cows with high and low NUE are presented in Table 6. The concentrations of total EAAs and total AAs were significantly lower in the high-NUE group than in the low-NUE group (*p* < 0.05). Among EAAs, concentrations of arginine, lysine, leucine, valine, and isoleucine were significantly lower or tended to be lower in cows with high NUE compared with those with low NUE (*p* ≤ 0.05), whereas no significant differences were observed in histidine, methionine, phenylalanine, or threonine concentrations between the two groups (*p* > 0.05). The concentration of total non-essential amino acids (NEAAs) did not differ significantly between the two groups (*p* > 0.05). Similarly, no significant differences were detected in the concentrations of individual NEAAs, including alanine, aspartate, cysteine, glutamate, glycine, proline, serine, or tyrosine (*p* > 0.05).

### 3.7. Milk Hydrolyzed Amino Acid Composition

Comparisons of milk hydrolyzed amino acid composition between cows with high and low NUE are presented in Table 7. The proportions of total EAAs and total NEAAs in milk protein did not differ significantly between the two groups (*p* > 0.05). Among EAAs, the proportion of methionine was significantly lower in the high-NUE group than in the low-NUE group (*p* = 0.003), whereas no significant differences were observed in arginine, histidine, isoleucine, leucine, lysine, phenylalanine, threonine, or valine proportions (*p* > 0.05). With respect to NEAAs, the proportion of alanine was significantly lower in cows with high NUE compared with those with low NUE (*p* = 0.036), and cysteine proportion tended to be lower in the high-NUE group, although this difference did not reach statistical significance (*p* = 0.066). No significant differences were detected in the proportions of aspartate, glutamate, glycine, proline, serine, or tyrosine between the two groups (*p* > 0.05).

### 3.8. Residual NUE Analysis Adjusted for Production Level

Multiple linear regression analysis showed that ECM and DMI together explained a substantial proportion of the variation in conventional NUE (R^2^ = 0.714, adjusted R^2^ = 0.710). The overall regression model was significant (F = 209.414, *p* < 0.001). Both DMI and ECM were significant predictors of NUE. DMI was negatively associated with NUE (unstandardized B = −0.010, standardized β = −0.881, *p* < 0.001), whereas ECM was positively associated with NUE (unstandardized B = 0.005, standardized β = 0.867, *p* < 0.001). No multicollinearity concern was detected between ECM and DMI, as indicated by tolerance values of 0.716 and VIF values of 1.397. The Kolmogorov-Smirnov test with Lilliefors correction indicated that the unstandardized residuals did not significantly deviate from normality (*p* = 0.200). Visual inspection of the normal Q-Q plot showed that most residuals were close to the reference line, although a small number of observations deviated in the lower tail. After excluding the observation with the most extreme standardized residual, the directions of the regression coefficients remained consistent with those of the original model (after exclusion: R^2^ = 0.736, adjusted R^2^ = 0.733; for DMI: unstandardized B = −0.010, standardized β = −0.882, *p* < 0.001; for ECM: unstandardized B = 0.006, standardized β = 0.888, *p* < 0.001), indicating that the model interpretation was robust. Therefore, the unstandardized residuals from the original model were extracted and defined as rNUE for subsequent analyses.

The regression results for the associations between rNUE and rumen fermentation and serum parameters are summarized in Table 8. After adjusting for production level, ruminal MCP concentration remained positively associated with rNUE (standardized β = 0.572, *p* = 0.021), and ruminal NH_3_-N concentration was also positively related to rNUE (standardized β = 0.663, *p* = 0.005). In addition, the acetate-to-propionate ratio was negatively associated with rNUE (standardized β = −0.540, *p* = 0.031). In contrast, ruminal pH, acetate concentration, and propionate concentration were not significantly related to rNUE (*p* > 0.10). No significant associations were observed between rNUE and serum total protein, albumin, globulin, blood urea nitrogen, glucose, total cholesterol, triglycerides, lactate dehydrogenase, or total antioxidant capacity (*p* > 0.10). Residual NUE was negatively associated with circulating amino acid concentrations. Specifically, total EAAs (standardized β = −0.422, *p* = 0.016), total NEAAs (standardized β = −0.367, *p* = 0.039), and total amino acids (standardized β = −0.462, *p* = 0.008) remained significantly associated with rNUE after adjustment. These findings indicate that cows with higher adjusted efficiency exhibited lower circulating amino acid pools.

## 4. Discussion

The present study demonstrates that dairy cows with divergent nitrogen use efficiency (NUE) exhibit coordinated differences in nitrogen partitioning, rumen fermentation, circulating amino acid profiles, and metabolic status, even under tightly controlled conditions of intake, parity, and lactation stage. High-NUE cows were characterized by greater milk yield and milk nitrogen output, increased ruminal microbial protein concentration, a lower acetate-to-propionate ratio, and reduced circulating amino acid concentrations. These findings confirm that NUE represents an integrated, whole-animal trait reflecting interactions among digestive, metabolic, and productive processes rather than a single physiological function.

### 4.1. Residual NUE as a Mathematical Framework for Evaluating Production-Adjusted Nitrogen Use Efficiency

Conventional NUE is calculated as the ratio of milk nitrogen output to nitrogen intake; therefore, it is inherently influenced by milk production and feed intake. Because milk nitrogen output is largely determined by milk yield and milk protein secretion, cows with greater milk production may exhibit higher apparent NUE even if their intrinsic metabolic efficiency is not necessarily improved. Thus, conventional NUE reflects both production-driven nitrogen partitioning and biological nitrogen utilization, which complicates the interpretation of efficiency differences among cows [10,41]. In practical dairy production, this limitation may lead to overestimation of nitrogen efficiency in high-producing cows and underestimation of cows with moderate production but potentially favorable nitrogen partitioning [42]. Similarly, in individual cow selection or breeding programs, reliance on conventional NUE alone may preferentially select animals with higher milk output rather than cows with genuinely improved production-adjusted nitrogen utilization. Therefore, conventional NUE should be interpreted cautiously and, where possible, complemented with production-adjusted indicators such as rNUE.

The present results support this concern. High-NUE cows had greater milk yield and milk nitrogen output than low-NUE cows, whereas nitrogen intake was similar between groups. Moreover, ECM and dry matter intake explained 71.4% of the variation in NUE, indicating that conventional NUE was largely driven by production level and feed intake. Therefore, high observed NUE should not be interpreted solely as evidence of superior intrinsic nitrogen metabolism.

To separate production-related effects from production-adjusted variation in nitrogen utilization, residual NUE was calculated as the deviation from expected NUE after accounting for ECM and dry matter intake. This approach is conceptually similar to RFI, in which feed intake is adjusted for production and maintenance requirements to identify inherent differences in feed efficiency. The lack of correlation between residual NUE and ECM indicated that the variation in NUE directly explained by ECM was statistically accounted for in the present model, rather than proving that all production-related effects were completely removed. Importantly, residual NUE remained associated with ruminal microbial crude protein, ruminal ammonia nitrogen, the acetate-to-propionate ratio, and circulating amino acid concentrations. These associations suggest that residual NUE captured biologically meaningful variation in nitrogen metabolism after adjustment for ECM and DMI. However, the rNUE model used in this study included only ECM and DMI and did not account for other potentially relevant factors, such as body weight, body condition score, energy balance, maintenance requirements, or digestive efficiency. Therefore, rNUE should be interpreted as a production-adjusted approximation of NUE rather than a complete measure of intrinsic nitrogen metabolism. In addition, because this model was developed using early-lactation Chinese Holstein cows fed a single diet on one commercial farm, its applicability across different breeds, lactation stages, dietary conditions, and management systems requires further validation and model recalibration. Therefore, residual NUE may provide a useful mathematical phenotype for evaluating production-adjusted individual variation in NUE under comparable feeding and management conditions in dairy cows.

### 4.2. Nitrogen Partitioning Under Divergent Efficiency Phenotypes

From a nitrogen partitioning perspective, the higher NUE in the H group was mainly attributable to greater milk nitrogen output under comparable nitrogen intake. However, urinary nitrogen excretion was also higher in these cows, indicating that increased milk nitrogen output was accompanied by greater overall nitrogen flux rather than simply reduced nitrogen loss. This interpretation is supported by the higher ruminal NH_3_-N and microbial crude protein concentrations observed in high-NUE cows, together with lower circulating total and essential amino acid concentrations, which may indicate greater ruminal nitrogen turnover and amino acid utilization for milk protein synthesis. Physiologically, ruminal ammonia not incorporated into microbial protein can be absorbed through the rumen wall, converted to urea in the liver, and then either recycled to the gastrointestinal tract or excreted in urine [43]. Therefore, the higher urinary nitrogen excretion observed in high-NUE cows may reflect increased nitrogen turnover associated with greater metabolic throughput [25,44,45]. However, because hepatic urea synthesis and urea recycling were not directly measured, this interpretation should be considered a plausible explanation rather than direct causal evidence. These findings emphasize that greater nitrogen excretion does not necessarily contradict higher NUE when considered in the context of increased production, but rather reflects altered partitioning of nitrogen among metabolic pathways.

### 4.3. Rumen Nitrogen Metabolism Is Associated with Production-Adjusted Variation in NUE

Rumen fermentation plays a central role in determining nitrogen conversion efficiency in dairy cows, because dietary nitrogen must first be degraded, assimilated by rumen microorganisms, and converted into microbial protein before contributing to intestinal amino acid supply [46]. In the present study, high-NUE cows had greater ruminal microbial crude protein concentration than low-NUE cows. Because MCP concentration is a static measurement rather than a direct measurement of microbial protein synthesis rate, this result should not be interpreted as direct evidence of enhanced microbial nitrogen synthesis efficiency [47]. Instead, it suggests that high-NUE cows had a larger ruminal microbial protein pool at the sampling time point, which may reflect greater microbial nitrogen capture or more favorable rumen conditions for microbial biomass accumulation [29,48]. This interpretation is consistent with previous studies reporting that improved ruminal nitrogen utilization and microbial protein formation are closely associated with dietary nitrogen availability, energy–nitrogen synchrony, and milk protein production in dairy cows [29,47]. However, because microbial protein flow to the intestine and dynamic microbial synthesis efficiency were not directly measured in the present study, the association between ruminal MCP concentration and rNUE should be interpreted as correlative rather than causal.

Interestingly, ruminal ammonia nitrogen concentration was also higher in high-NUE cows than in low-NUE cows. Ruminal ammonia nitrogen is an essential nitrogen source for microbial protein synthesis, and concentrations below approximately 5 mg/dL are generally considered insufficient to support optimal microbial growth [49]. In the present study, ruminal ammonia nitrogen concentrations in both groups were above this commonly cited threshold, with values of 13.24 and 11.14 mg/dL in high- and low-NUE cows, respectively. These values suggest that ammonia nitrogen availability was unlikely to be limiting in either group [50]. However, the higher ruminal ammonia nitrogen concentration in high-NUE cows should be interpreted cautiously, because elevated ruminal ammonia nitrogen may have two possible implications. On the one hand, it may provide sufficient nitrogen substrate for microbial growth when fermentable energy is available; on the other hand, it may also reflect an imbalance between ruminal nitrogen degradation and microbial nitrogen capture, potentially increasing ammonia absorption and urinary nitrogen excretion [51,52].

In the present study, the simultaneous increases in ruminal ammonia nitrogen and microbial crude protein concentrations in high-NUE cows suggest that greater ammonia availability was accompanied by a larger ruminal microbial protein pool at the sampling time point. Nevertheless, because microbial crude protein concentration is a static indicator and microbial protein flow or synthesis efficiency was not directly measured, this result should not be interpreted as direct evidence of enhanced microbial nitrogen synthesis. Moreover, urinary nitrogen excretion was also greater in high-NUE cows, indicating that part of the increased ruminal ammonia may have been absorbed, converted to urea, and excreted in urine. Therefore, higher ruminal ammonia nitrogen in high-NUE cows may reflect both greater ruminal nitrogen availability and increased nitrogen turnover, rather than solely beneficial microbial nitrogen utilization.

After accounting for ECM and dry matter intake, residual NUE remained positively associated with both ruminal ammonia nitrogen and microbial crude protein concentrations. This finding suggests that ruminal nitrogen metabolism may represent an important metabolic node associated with production-adjusted variation in NUE. However, because the present study was observational, these associations should be interpreted as correlative rather than causal.

The lower acetate-to-propionate ratio observed in high-NUE cows further indicates a shift in rumen fermentation pattern. In ruminant nutrition, a lower acetate-to-propionate ratio generally reflects relatively greater glucogenic fermentation, because propionate is the major ruminal precursor for hepatic gluconeogenesis [53,54,55]. Increased propionate supply may support glucose availability for lactose synthesis, which is closely linked to milk volume, while also providing fermentable energy required for microbial growth and microbial protein formation [50]. Because microbial protein synthesis requires both nitrogen substrates and fermentable energy, a lower acetate-to-propionate ratio may indicate a ruminal environment more favorable for coupling nitrogen release with microbial nitrogen capture [56]. Importantly, the negative association between residual NUE and the acetate-to-propionate ratio suggests that this fermentation pattern was linked to production-adjusted variation in NUE after accounting for ECM and DMI, rather than merely being a consequence of higher milk yield. From a practical perspective, this finding suggests that dietary strategies aimed at improving NUE should not focus only on crude protein supply, but should also consider rumen-fermentable carbohydrate availability and energy–nitrogen synchrony. However, because ruminal fermentation was measured at a single time point and dietary treatments were not imposed, this interpretation should be regarded as associative rather than causal.

Under practical feeding conditions, the greatest deviations from the present findings would likely occur when dietary protein supply and fermentable energy availability are not properly balanced. For example, excess crude protein or rumen-degradable protein may increase ruminal ammonia accumulation, hepatic urea formation, and urinary nitrogen excretion, whereas insufficient rumen-degradable protein or fermentable carbohydrate supply may limit microbial protein formation and impair energy–nitrogen synchrony. Marked changes in starch-to-fiber balance or physically effective fiber may also alter volatile fatty acid profiles, particularly the acetate-to-propionate ratio, thereby affecting glucogenic energy supply, microbial growth, and milk synthesis.

Overall, these findings indicate that rumen fermentation traits, particularly microbial crude protein synthesis, ammonia nitrogen availability, and the acetate-to-propionate ratio, are closely associated with the intrinsic component of NUE. However, because rumen fluid was collected from a subset of cows and the present study did not directly measure ruminal microbial community structure or nitrogen flow to the small intestine, these relationships should be interpreted as associative rather than causal. Further studies integrating rumen microbiome, nitrogen flux, and microbial protein flow measurements are needed to clarify the mechanisms by which rumen nitrogen metabolism contributes to residual NUE.

### 4.4. Amino Acid Utilization and Metabolic Turnover

Alterations in circulating amino acid profiles provide additional insight into metabolic efficiency. The lower serum concentrations of total and essential amino acids observed in high-NUE cows, despite greater milk protein output, may suggest an increased rate of amino acid utilization and clearance for milk synthesis [57,58,59]. However, lower circulating amino acid concentrations may also be influenced by other processes, including differences in intestinal amino acid absorption and accelerated whole-body amino acid turnover; therefore, they should not be interpreted solely as evidence of enhanced mammary amino acid uptake. Consistently, Li et al. [60] reported that in primiparous dairy cows, high-NUE animals exhibited 12.6% lower total plasma amino acid concentrations and significantly lower levels of individual amino acids, including arginine, glutamine, leucine, lysine, and ornithine, while producing 6.2 kg more milk per day. The persistence of these associations with rNUE further suggests that circulating amino acid pools were related to production-adjusted variation in NUE after accounting for ECM and DMI, but does not directly demonstrate enhanced amino acid uptake by peripheral tissues or the mammary gland. In contrast, higher circulating amino acid concentrations in low-NUE cows may reflect lower amino acid utilization, altered intestinal amino acid supply, or differences in whole-body amino acid turnover, rather than simply accumulation in the bloodstream.

The stability of milk protein amino acid composition between groups suggests strong homeostatic regulation of mammary protein synthesis, despite substantial differences in systemic amino acid availability [61,62]. This is supported by the finding that milk protein content did not differ between high- and low-NUE cows, despite marked differences in circulating amino acid profiles [60]. In the present study, the largely unchanged milk hydrolyzed amino acid profile, together with lower circulating amino acid concentrations in high-NUE cows, suggests that differences in serum amino acid pools were more closely related to amino acid availability and utilization dynamics than to major changes in milk protein amino acid composition. Minor changes in specific amino acids, such as methionine and alanine, may reflect differential partitioning between milk synthesis and other metabolic pathways, including methyl metabolism and gluconeogenesis [63,64,65,66,67]. The persistence of these associations with rNUE suggests that amino acid metabolism may be an important component associated with production-adjusted NUE, although direct measurements of intestinal amino acid absorption, mammary amino acid uptake, and whole-body amino acid turnover are needed to confirm this mechanism.

### 4.5. Metabolic Load and Oxidative Status

With respect to oxidative status, the lower total antioxidant capacity observed in high-NUE cows was more likely related to their greater production level and associated metabolic load rather than intrinsic efficiency, as the association disappeared after adjustment. This interpretation is consistent with previous studies showing that high-producing lactating cows are more susceptible to oxidative challenge because elevated milk synthesis increases energy demand, mitochondrial oxidative metabolism, and reactive oxygen species generation [68,69]. Under these conditions, antioxidant compounds and antioxidant enzyme systems may be increasingly consumed to counteract oxidative pressure, resulting in reduced circulating total antioxidant capacity [70]. This lack of association with rNUE indicates that oxidative status is linked to metabolic load rather than intrinsic nitrogen use efficiency. In the present study, total antioxidant capacity was lower in high-NUE cows before adjustment, but it was not associated with rNUE after accounting for ECM and DMI. Therefore, oxidative status appeared to reflect production-related metabolic load rather than production-adjusted variation in NUE. However, because only total antioxidant capacity was measured, and specific oxidative stress markers or antioxidant enzymes were not comprehensively evaluated, this interpretation should be considered preliminary.

### 4.6. Limitations and Implications

Several limitations should be considered. First, urinary nitrogen was estimated from spot samples, and apparent nitrogen balance does not directly quantify tissue protein deposition. Second, residual NUE was calculated using only ECM and DMI, without accounting for body weight, energy balance, or digestive efficiency; thus, it represents a partial approximation rather than a complete measure of intrinsic efficiency. Third, rumen fermentation variables were measured in a subset of cows, and key parameters such as microbial community structure, nutrient digestibility, and mammary amino acid uptake were not directly determined, and the observed associations between rNUE and metabolic indicators should be interpreted as correlative rather than causal. Fourth, the present study was conducted over a relatively short period at a single commercial dairy farm using a single diet and early-lactation Holstein cows. Holstein cows were selected because they are the predominant high-yielding dairy breed used in commercial milk production systems in China, making them a relevant population for studying NUE under Chinese dairy production conditions. However, the applicability of the present findings to other breeds, lactation stages, farms, dietary systems, and geographic regions requires further validation. Accordingly, the current rNUE framework is most appropriate for screening individual cows managed under similar feeding and production conditions, and it should not be directly used for comparative evaluation across farms, breeds, or markedly different diets without model recalibration. Future studies should validate this framework in larger multi-farm populations, establish dietary crude protein or rumen-degradable protein gradients, conduct long-term repeated measurements across lactation stages, and evaluate the repeatability and genetic stability of rNUE-related traits.

## 5. Conclusions

In conclusion, this study demonstrates that nitrogen use efficiency in dairy cows is largely driven by milk production levels, with ECM and DMI explaining 71.4% of its variation. However, by applying a residual-based approach, we identified a production-adjusted component of NUE that reflects intrinsic metabolic efficiency. This residual efficiency was associated with ruminal nitrogen metabolism and amino acid utilization, indicating biologically meaningful variation beyond milk yield. These findings suggest that conventional NUE metrics may overestimate efficiency differences driven by production and highlight the importance of separating production and metabolic components when evaluating nitrogen efficiency in dairy systems.

## Figures and Tables

**Table 1:** Ingredients and chemical composition (% of DM) of experimental diets fed to all cows.

Ingredient	% of DM	Chemical Composition	% of DM
Corn silage	30.05	DM, % as-fed	50.55
Dry ground corn grain	24.34	Crude Protein	17.20
Alfalfa hay	12.00	Ether Extract	5.50
Corn flakes	6.06	Neutral Detergent Fiber	32.50
Cottonseed	4.45	Acid Detergent Fiber	19.55
Oaten hay	3.63	Ash	7.50
Sugar beet meal	3.63	Ca	0.79
Rapeseed meal	3.28	P	0.41
Brewers grain	3.07	GE (Mcal/kg of DM)	4.51
Dried distillers grains with solubles	2.73	NE_L_ (Mcal/kg of DM)	1.75
Soybean meal RUP source	1.77		
Barley	1.37		
Puffing of soybean	1.34		
NaHCO_3_	0.78		
Calcium salts of fatty acids	0.75		
Premix ^1^	0.75		

^1^ The premix provided the following nutrients per kilogram of diet: vitamin A, 5130 IU; vitamin D_3_, 1283 IU; vitamin E, 26 mg; biotin, 0.05 mg; β-carotene, 0.10 mg; manganese, 12 mg; phosphorus, 12 mg; sulfur, 0.85 mg; zinc, 64 mg; selenium, 0.40 mg; and cobalt, 0.19 mg. GE—gross energy; NE_L_—net energy for lactation, calculated according to NRC (2001) [36]. RUP—rumen-undegradable protein.

**Table 2:** Comparison of body measurement traits between cows with high and low nitrogen use efficiency.

	Treatment			
Items	H	L	SEM	*p*-Value
Body height, cm	148.38	148.31	0.497	0.690
Chest circumference, cm	213.31	214.06	1.553	0.806
Body length, cm	165.63	170.25	1.355	0.088
Body weight ^1^, kg	697.35	720.89	12.661	0.361

^1^ Body weight was estimated using an empirical equation based on heart girth and body length as follows: body weight (kg) = (heart girth^2^ × body length)/10,840.

**Table 3:** Comparison of lactation performance between cows with high and low nitrogen use efficiency.

	Treatment			
Items	H	L	SEM	*p*-Value
Parity	2.13	2.44	0.175	0.517
Days in milk, d	63.00	68.13	2.825	0.373
Dry matter intake, kg/d	25.19	26.45	0.713	0.384
Milk yield, kg/d	44.25	33.44	1.350	<0.001
Milk protein, %	3.22	3.34	0.017	<0.001
Milk protein yield, kg/d	1.42	1.11	0.040	<0.001
Milk fat, %	3.51	4.15	0.089	<0.001
Milk fat yield, kg/d	1.55	1.38	0.049	0.081
Lactose, %	4.88	4.98	0.020	0.014
Total solids, %	12.51	13.37	0.108	<0.001
Milk urea nitrogen, mg/dL	11.37	13.55	0.394	0.004
Fat-to-protein ratio	1.09	1.24	0.025	0.001
SCC, 10^4^ cells/mL	9.18	9.42	1.140	0.985

**Table 4:** Comparison of rumen fermentation parameters between cows with high and low nitrogen use efficiency.

	Treatment			
Items	H	L	SEM	*p*-Value
MCP, mg/mL	0.80	0.68	0.019	<0.001
pH	6.45	6.50	0.034	0.475
NH_3_-N, mg/dL	13.24	11.14	0.431	0.009
Acetate, mmol/L	50.23	51.82	1.954	0.699
Propionate, mmol/L	25.27	22.24	0.820	0.062
Isobutyric acid, mmol/L	0.51	0.45	0.038	0.463
Butyrate, mmol/L	10.46	9.06	0.434	0.109
Isovaleric acid, mmol/L	0.85	0.82	0.076	0.810
Valeric acid, mmol/L	1.15	0.90	0.076	0.098
Total volatile fatty acid, mmol/L	88.58	85.14	2.826	0.561
Acetate/Propionate ratio	2.01	2.33	0.081	0.042

**Table 5:** Comparisons of nitrogen partitioning between cows with high and low nitrogen use efficiency.

	Treatment			
Items	H	L	SEM	*p*-Value
N intake, g/d	693.15	727.93	19.618	0.384
Milk N, g/d	222.92	174.64	6.262	<0.001
NUE, %	32.49	24.05	0.892	<0.001
Urinary N, g/d	242.33	211.60	7.330	0.034
Urinary N, %	35.14	29.27	0.893	<0.001
Fecal N, g/d	197.78	201.12	6.068	0.788
Fecal N, %	28.58	27.75	0.587	0.490
Apparent N balance ^1^, g/d	30.12	140.57	13.452	<0.001

^1^ Apparent N balance was calculated as follows: apparent N balance = N intake—milk N—urinary N—fecal N.

**Table 6:** Comparisons of serum-free amino acid concentrations between cows with high and low nitrogen use efficiency.

	Treatment			
Items, mg/L	H	L	SEM	*p*-Value
Total EAA	156.13	182.78	5.197	0.008
Arg	14.28	20.33	0.928	<0.001
His	9.06	9.61	0.388	0.484
Ile	17.08	19.40	0.596	0.050
Leu	22.40	26.09	0.945	0.049
Lys	13.26	16.22	0.640	0.018
Met	3.65	3.93	0.161	0.399
Phe	9.21	9.54	0.299	0.587
Thr	34.12	37.92	2.079	0.370
Val	34.95	39.73	1.193	0.043
Total NEAA	94.17	100.27	2.421	0.213
Ala	22.63	22.10	0.905	0.778
Asp	1.31	1.44	0.099	0.534
Cys	2.77	2.51	0.244	0.600
Glu	17.87	20.31	1.314	0.362
Gly	21.74	22.72	0.908	0.598
Pro	8.31	9.56	0.430	0.149
Ser	8.30	9.30	0.396	0.213
Tyr	11.24	12.33	0.539	0.320
Total AA	250.30	283.05	6.675	0.012

EAA—essential amino acid; Arg—arginine; His—histidine; Ile—isoleucine; Leu—leucine; Lys—lysine; Met—methionine; Phe—phenylalanine; Thr—threonine; Val—valine; NEAA—non-essential amino acids; Ala—alanine; Asp—aspartic acid; Cys—cysteine; Glu—glutamic acid; Gly—glycine; Pro—proline; Ser—serine; Tyr—tyrosine.

**Table 7:** Comparisons of milk hydrolyzed amino acid composition between cows with high and low nitrogen use efficiency.

	Treatment			
Items, %	H	L	SEM	*p*-Value
EAA	48.36	48.43	0.046	0.489
Arg	3.24	3.25	0.007	0.338
His	2.95	2.93	0.012	0.313
Ile	5.35	5.38	0.010	0.083
Leu	9.99	10.02	0.017	0.323
Lys	8.72	8.69	0.020	0.339
Met	2.30	2.39	0.014	0.003
Phe	5.05	5.00	0.019	0.187
Thr	4.30	4.30	0.010	0.877
Val	6.47	6.48	0.010	0.439
NEAA	51.64	51.57	0.046	0.489
Ala	3.26	3.31	0.013	0.036
Asp	7.64	7.60	0.016	0.278
Cys	0.65	0.74	0.024	0.066
Glu	20.20	20.03	0.060	0.163
Gly	1.82	1.82	0.007	0.754
Pro	9.75	9.81	0.024	0.198
Ser	4.99	4.99	0.017	0.972
Tyr	3.32	3.26	0.030	0.319

EAA—essential amino acid; Arg—arginine; His—histidine; Ile—isoleucine; Leu—leucine; Lys—lysine; Met—methionine; Phe—phenylalanine; Thr—threonine; Val—valine; NEAA—non-essential amino acids; Ala—alanine; Asp—aspartic acid; Cys—cysteine; Glu—glutamic acid; Gly—glycine; Pro—proline; Ser—serine; Tyr—tyrosine.

**Table 8:** Associations between residual NUE and rumen fermentation and serum parameters.

Dependent Variable	β (Standardized)	*p*-Value	R^2^
MCP	0.57	0.021	0.327
NH_3_-N	0.66	0.005	0.440
Acetate/Propionate ratio	−0.54	0.031	0.292
Total EAA	−0.42	0.016	0.178
Total NEAA	−0.37	0.039	0.135
Total AA	−0.46	0.008	0.213
Total antioxidant capacity	−0.17	0.357	0.019

R^2^ refers to the coefficient of determination from the linear regression model.

## Data Availability

The original contributions presented in this study are included in the article. Further inquiries can be directed to the corresponding author.

## Abbreviations

The following abbreviations are used in this manuscript:

NUE	Nitrogen use efficiency
DM	Dry matter
DMI	Dry matter intake
TMR	Total mixed ration
EE	Ether extract
NDF	Neutral detergent fiber
ADF	Acid detergent fiber
GE	Gross energy
NE_L_	Net energy for lactation
RUP	Rumen-undegradable protein
MCP	Microbial crude protein
VFA	Volatile fatty acid
NH_3_-N	Ammonia nitrogen
SCC	Somatic cell count
SEM	Standard error of the mean
EAA	Essential amino acid
NEAA	Non-essential amino acid
AA	Amino acids
Arg	Arginine
His	Histidine
Ile	Isoleucine
Leu	Leucine
Lys	Lysine
Met	Methionine
Phe	Phenylalanine
Thr	Threonine
Val	Valine
Ala	Alanine
Asp	Aspartic acid
Cys	Cysteine
Glu	Glutamic acid
Gly	Glycine
Pro	Proline
Ser	Serine
Tyr	Tyrosine
